# Copper ferrite–graphene oxide catalyst for enhanced peroxymonosulfate activation and pollutant degradation

**DOI:** 10.1039/d5na00409h

**Published:** 2025-08-11

**Authors:** Imane Sebah, Moustapha Belmouden

**Affiliations:** a Laboratory of Organic Chemistry and Physical Chemistry (Molecular Modeling and Environment), Faculty of Sciences, University Ibn Zohr Agadir Morocco Imane.sabah@outlouk.fr

## Abstract

In this study, a magnetic nanocomposite of copper ferrite (CuFe_2_O_4_) supported on reduced graphene oxide (rGO) was synthesized *via* a solvothermal method and applied as a catalyst for the activation of peroxymonosulfate (PMS) to degrade Orange G (OG) dye in aqueous solution. The structure and morphology of the catalyst were thoroughly characterized using XRD, FTIR, SEM, STEM, and nitrogen adsorption–desorption analyses. The rGO/CuFe_2_O_4_ composite demonstrated superior catalytic performance, achieving 90.8% OG removal within 60 minutes, attributed to its enhanced surface area, efficient radical generation, and strong interaction between rGO and CuFe_2_O_4_. The system exhibited high activity across a wide pH range, significant mineralization (78% TOC removal), and good recyclability over four cycles. The catalyst also effectively degraded other dyes including rhodamine B (78%), methylene blue (86%), and methyl orange (89%) under similar conditions. These findings suggest that rGO/CuFe_2_O_4_ is a promising, reusable catalyst for advanced oxidation processes in wastewater treatment.

## Abstract 2

The rGO/CuFe_2_O_4_ catalyst activates peroxymonosulfate to generate reactive oxygen species (˙OH and SO_4_˙^−^), enabling oxidative degradation of organic pollutants into CO_2_ and H_2_O.

## Graphical abstract

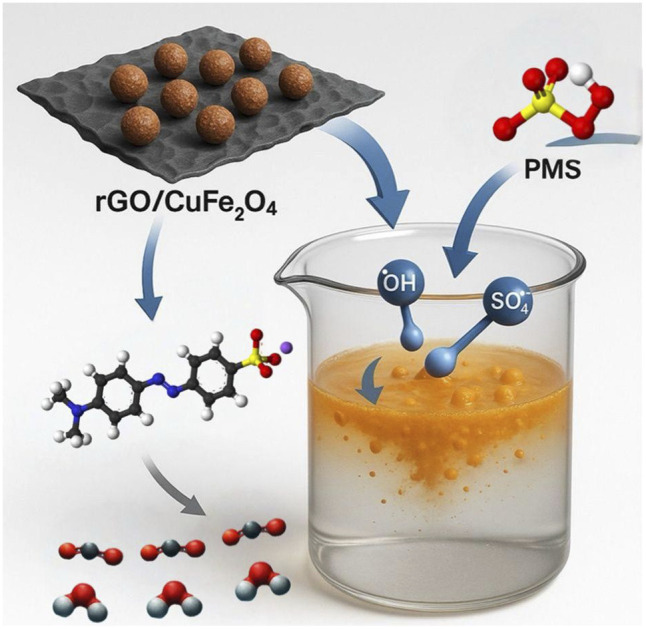

## 1. Introduction

The demand for effective water treatment technologies is increasing due to the rising discharge of wastewater containing persistent organic pollutants, which pose significant threats to ecosystems and public health.^1^ Among these, synthetic dyes used in various industries—particularly textiles—are of special concern, environmentally persistent, toxic, and often carcinogenic and mutagenic.^2^ Because of their complex molecular structures and high stability against natural degradation, effective removal of these pollutants from water bodies is urgently required.^3,4^

Among the various chemical approaches to wastewater treatment,^5–7^ the Fenton reaction has long been recognized for its high efficiency in degrading recalcitrant organic contaminants. This classical process, based on the reaction between ferrous ions (Fe^2+^) and hydrogen peroxide (H_2_O_2_), leads to the *in situ* formation of hydroxyl radicals (˙OH), which possess strong oxidative potential and can mineralize a wide spectrum of pollutants.^8^ However, limitations such as strict pH requirements (typically around pH 3), iron sludge generation, and reduced activity in neutral conditions have prompted the development of alternative processes.^9^ In this context, sulfate radical-based advanced oxidation processes (SR-AOPs), which rely on the activation of persulfate (PS, S_2_O_8_^2−^) or peroxymonosulfate (PMS, HSO_5_^−^), have gained increasing attention due to the high redox potentials, longer lifetime, and broader pH tolerance of sulfate radicals (SO_4_˙^−^) compared to ˙OH radicals.^10–14^ The SO_4_˙^−^ radicals exhibit a longer half-life (30–40 μs), broader pH adaptability (2–9), and a comparable redox potential (2.5–3.1 V *vs.* NHE), making SR-AOPs especially attractive for wastewater treatment.^12,15–18^

To activate PS or PMS, various techniques have been explored, including UV irradiation, ultrasound, electrochemical methods, thermal activation, and transition metal-based catalysis.^19–22^ For instance, Hassani *et al.*^21^ presented a comprehensive review on ultrasound-assisted oxidant systems, highlighting how acoustic cavitation can enhance PS/PMS activation by promoting the formation of reactive radicals and improving mass transfer. Electrochemical methods have also gained attention for their controllability and efficiency in generating radicals under mild conditions.^23^ Among these strategies, transition metal-based catalysts have garnered significant interest due to their ability to facilitate redox reactions and generate reactive radicals. In particular, bimetallic systems and metal oxides, such as Co^2+^, Fe^2+^, Cu^2+^, Mn^2+^, and their oxides (*e.g.*, Co_3_O_4_, Fe_3_O_4_, CuO, MnO_2_), have shown strong catalytic activity in PS/PMS activation.^24–28^ Among various catalysts, magnetic spinel ferrites, combining multiple transition metals in a stable structure, have emerged as effective materials for activating persulfate-based oxidants, owing to their redox activity, chemical robustness, and magnetic properties that facilitate recovery.^29^ Among them, cobalt ferrite (CoFe_2_O_4_) has been extensively studied and shows excellent catalytic efficiency in PMS activation. However, the potential leaching of toxic Co^2+^ ions raises environmental concerns, prompting the search for safer alternatives.^29^ Copper ferrite (CuFe_2_O_4_) has emerged as a promising substitute due to its low cost, environmental compatibility, and good catalytic performance. It has been successfully applied in the degradation of various organic pollutants, including dyes, pesticides and phenolic compounds, *via* PMS activation.^30–32^ Nevertheless, the catalytic efficiency of bulk CuFe_2_O_4_ is still limited by a relatively low surface area, limited conductivity, and particle aggregation, which can hinder electron transfer and reduce active site accessibility.

To overcome these drawbacks, integrating CuFe_2_O_4_ with conductive carbon-based supports has been proposed. Reduced graphene oxide (rGO), in particular, offers a large surface area, good electrical conductivity, and abundant defect sites (*e.g.*, vacancies, edge planes) that can serve as additional active sites for persulfate activation.^33–35^ The rGO/CuFe_2_O_4_ composite not only enhances the catalytic activity by improving electron transport and pollutant accessibility but also retains magnetic properties for easy recovery and reuse. Moreover, the spinel structure of CuFe_2_O_4_ provides chemical stability, reducing the risk of metal leaching.

Herein, we report the synthesis of rGO-supported CuFe_2_O_4_*via* a solvothermal method and assess its efficacy as a heterogeneous catalyst for PMS activation. The prepared materials were thoroughly characterized, and their catalytic performance is evaluated for the degradation of orange G dye. The effects of operational parameters, such as catalyst dosage, PMS concentration, and pH, were investigated, along with reusability and degradation efficiency toward other pollutants. This study aims to advance the development of environmentally friendly and high-performance catalysts for sustainable wastewater remediation.

## 2. Experimental section

### 2.1 Chemicals

Ferric chloride hexahydrate (FeCl_3_·6H_2_O, 99%), copper nitrate trihydrate (Cu(NO_3_)_2_·3H_2_O, 99%), ethylene glycol (EG, 98%), polyethylene glycol (PEG, *M*_w_ ≈ 1500), sodium acetate (NaAc, 99%), graphite powder (particle size ≤ 20 μm), hydrogen peroxide (H_2_O_2_, 30%), potassium permanganate (KMnO_4_), sodium nitrate (NaNO_3_), sulfuric acid (H_2_SO_4_, 98%), potassium peroxymonosulfate (OXONE®, 2KHSO_5_·KHSO_4_·K_2_SO_4_), l-histidine (99%), *para*-benzoquinone (*p*-BQ, 98%) were purchased from Sigma-Aldrich. Ethanol (EtOH) and *tert*-butyl alcohol (TBA) were obtained from Alfa Aesar. All samples were prepared from deionized water.

### 2.2 Synthesis of the CuFe_2_O_4_ and rGO–CuFe_2_O_4_

**Fig. 1:**
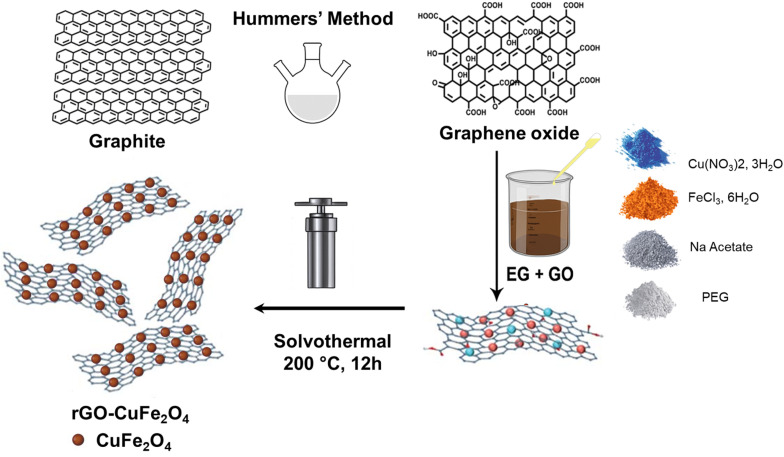
Schematic illustration of the hydrothermal synthesis of rGO/CuFe_2_O_4_ nanocomposite.

The rGO–CuFe_2_O_4_ nanocomposite was synthesized following a solvothermal method, as illustrated in Fig. 1. First, 0.15 g of graphene oxide (GO) (prepared using the Hummers' method)^36^ was dispersed in 20 mL of EG by ultrasonication for 1 hour to form a uniform suspension. Then, 0.24 g (0.001 mol) of Cu(NO_3_)_2_·3H_2_O, 0.54 g (0.002 mol) of FeCl_3_·6H_2_O, and 1 g (0.013 mol) of NaAc were added to the GO suspension and stirred at room temperature for 1 additional hour. The mixture was transferred into a Teflon-lined autoclave and heated at 200 °C for 12 hours. After cooling, the resulting product was separated using a magnet, washed thoroughly with deionized water and ethanol, and dried at 60 °C in a vacuum desiccator.

The parent CuFe_2_O_4_ material was synthesized using the same procedure without the addition of GO.

### 2.3 Catalytic testing

Orange G (OG) was employed as a model pollutant to evaluate the catalytic activity of CuFe_2_O_4_ and rGO/CuFe_2_O_4_ catalysts. The degradation experiments were conducted by dispersing a specific amount of catalyst and peroxymonosulfate (PMS) into 100 mL of OG solution (50 mg L^−1^) under continuous stirring. At predetermined time intervals, 5 mL aliquots were withdrawn and filtered through a 0.4 μm membrane to remove any residual catalyst. The concentration of OG in the treated samples was then determined using a UV-vis spectrophotometer by monitoring the absorbance at the characteristic wavelength of 484 nm.

The initial pH of the solution was adjusted using either 0.1 M NaOH or 0.1 M HCl, depending on the desired pH level. Furthermore, the catalytic performance of the rGO/CuFe_2_O_4_ system was evaluated against additional organic pollutants, including Rhodamine B (RhB), Methylene Blue (MB), and Methyl Orange (MO), under the same experimental conditions as those used for OG, with adjustments to the initial pollutant concentrations: [MO] = 50 mg L^−1^, [RhB] = 10 mg L^−1^, and [MB] = 10 mg L^−1^.

To assess the reusability of the rGO/CuFe_2_O_4_ catalyst, recyclability tests were carried out. After each degradation cycle, the catalyst was separated using an external permanent magnet, thoroughly washed with deionized water and ethanol, and then dried at 60 °C. The recovered catalyst was reused in subsequent degradation experiments under identical conditions, following the same procedure as described above.

### 2.4 Characterization

The structural properties of CuFe_2_O_4_ and rGO/CuFe_2_O_4_ were investigated using X-ray diffraction (XRD) with a Bruker D8 Advance Twin diffractometer, employing Cu Kα radiation (*λ* = 1.5418 Å) operated at 40 kV and 40 mA.

Fourier-transform infrared (FTIR) spectroscopy was performed in the spectral range of 4000–400 cm^−1^ using an ABB Bomem FTLA 2000 spectrometer (resolution: 4 cm^−1^) in KBr pellet mode, to identify functional groups and confirm interactions between the components.

Surface morphology and microstructure were analyzed by scanning electron microscopy (SEM) and scanning transmission electron microscopy (STEM) using a Tecnai G2 microscope, operated at an accelerating voltage of 120 kV.

The specific surface area and pore characteristics were determined by nitrogen adsorption–desorption isotherms using the Brunauer–Emmett–Teller (BET) method on a Micromeritics 3Flex surface analyzer. Prior to analysis, all samples were degassed at 250 °C for 12 hours.

Total Organic Carbon (TOC) analysis was performed using a Shimadzu TOC-L analyzer to evaluate the degree of mineralization during the degradation process. Samples were taken at various reaction times, filtered through a 0.22 μm membrane to remove solids, and acidified to pH < 2 to eliminate inorganic carbon before measurement. Calibration was carried out using potassium hydrogen phthalate standards to ensure accurate quantification of organic carbon.

The concentration of copper ions leached from rGO–CuFe_2_O_4_ was analyzed by inductively coupled plasma mass spectrometry (ICP-MS) using 7900 ICP-MS instrument.

### 2.5 Characterization of prepared materials

**Fig. 2:**
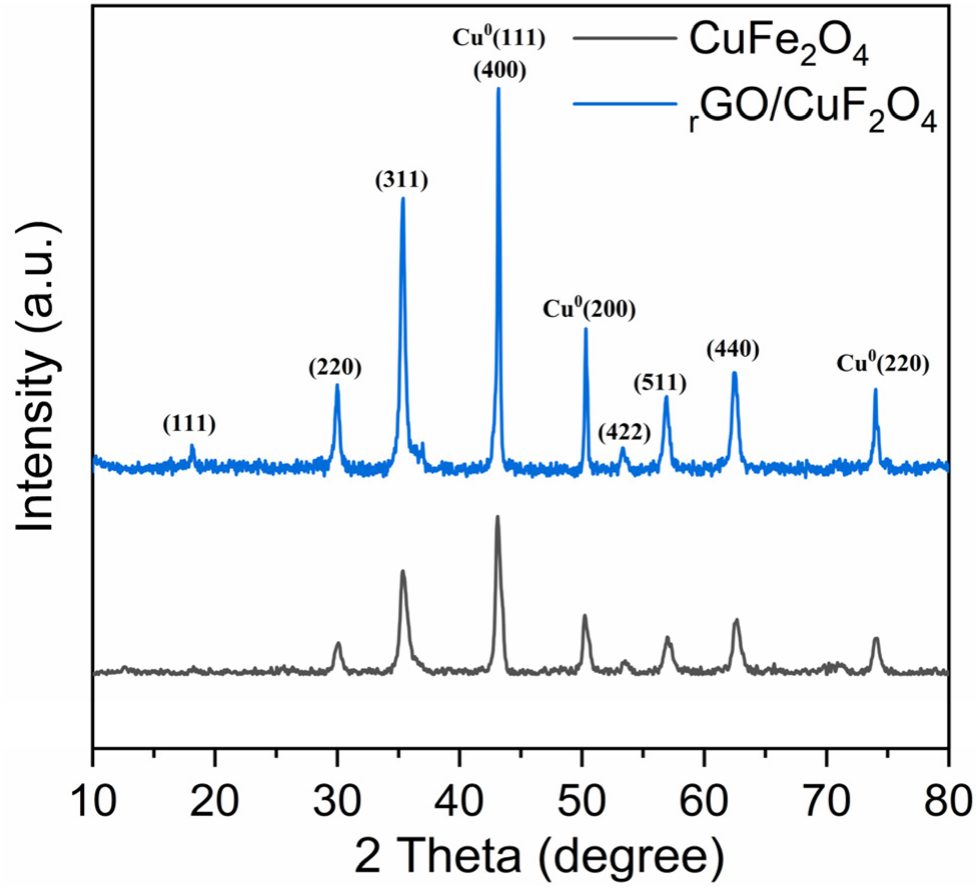
XRD patterns of CuFe_2_O_4_ and rGO/CuFe_2_O_4_ samples.

The XRD patterns of the obtained CuFe_2_O_4_ and the rGO/CuFe_2_O_4_ nanocomposite are presented in Fig. 2. The characteristic diffraction peaks of CuFe_2_O_4_, visible in both patterns, correspond to the spinel structure of copper ferrite. These peaks are indexed to the (111), (220), (311), (400), (422), (511), and (440) planes at 2*θ* values of approximately 18.3°, 29.9°, 35.4°, 43.1°, 53.3°, 56.9°, and 62.4°, respectively, which align well with the standard JCPDS card for CuFe_2_O_4_ (JCPDS no. 01-077-0010). This confirms the successful formation of crystalline CuFe_2_O_4_ in both samples. In addition, peaks at 2*θ* = 43.1°, 50.3°, and 74.1°, corresponding to the (111), (200), and (220) reflections of face-centered cubic metallic copper (Cu^0^) (JCPDS no. 01-85-1326), are observed in both samples. This indicates that partial reduction of Cu^2+^ to Cu^0^ occurred during the synthesis, facilitated by the presence of ethylene glycol, which serves as a strong reducing agent under high-temperature conditions. Given the low reduction potential of Cu^2+^ to Cu^0^ (+0.34 V), the reduction of Cu^2+^ ions in the solvothermal system is highly feasible. The presence of Cu^0^ in the synthesis of CuFe_2_O_4_ particles has also been previously reported in the literature.^37,38^

**Fig. 3:**
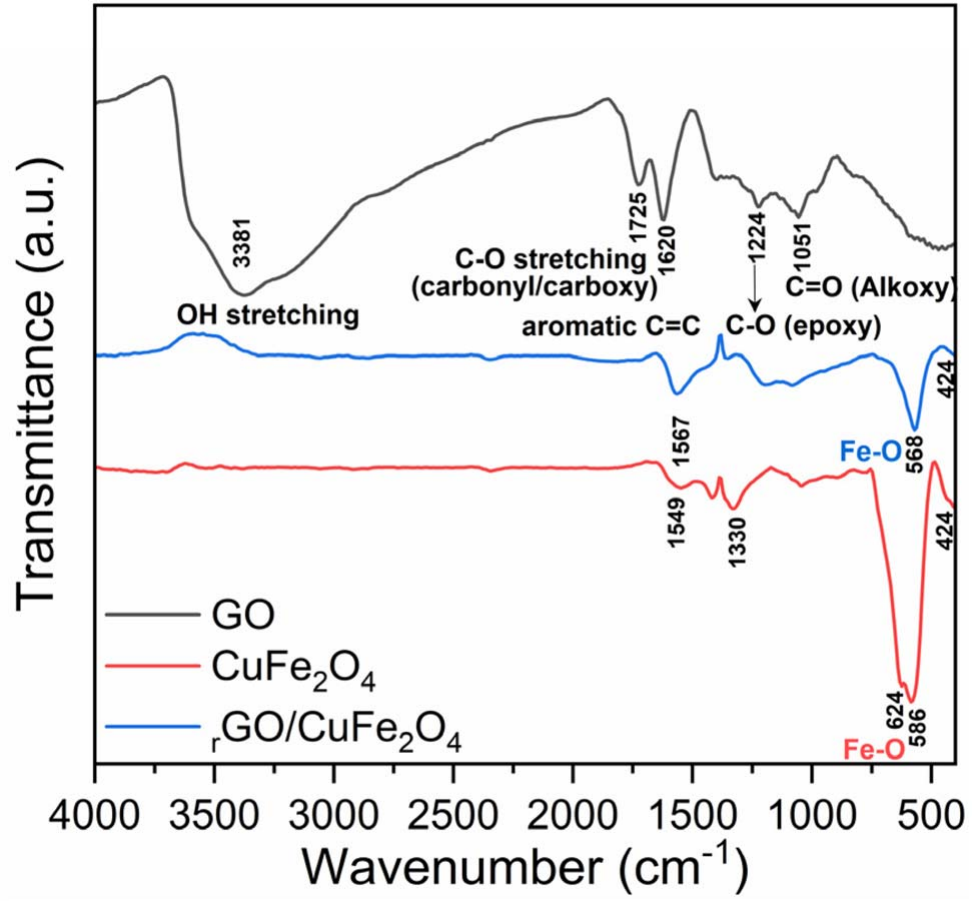
FTIR spectra of graphene oxide (GO), CuFe_2_O_4_ and rGO/CuFe_2_O_4_ samples.

The FTIR spectra of graphene oxide (GO), CuFe_2_O_4_, and the rGO/CuFe_2_O_4_ composite are shown in Fig. 3. The spectrum of GO displays a broad peak at 3381 cm^−1^, corresponding to O–H stretching vibrations from hydroxyl groups and adsorbed water. The presence of oxygen-containing functional groups is confirmed by the peak at 1725 cm^−1^, attributed to C=O stretching of carbonyl/carboxyl groups, and peaks at 1224 cm^−1^ and 1051 cm^−1^, corresponding to C–O stretching vibrations from epoxy and alkoxy groups, respectively.^39^ Additionally, the peak at 1620 cm^−1^ is assigned to the C=C stretching vibration, characteristic of the skeletal vibrations of unoxidized graphene domains.

For CuFe_2_O_4_, the spectrum exhibits strong peaks at 568 cm^−1^ and 624 cm^−1^, which can be attributed to Fe–O stretching vibrations in the octahedral site of the CuF_2_O_4_ crystal structure. The band at around 424 cm^−1^ can be attributed to the stretching vibration of Fe–O in the tetrahedral site of the CuF_2_O_4_ crystal structure.^40^ The peaks at 1549 cm^−1^ and 1330 cm^−1^ may correspond to O–H bending vibrations from adsorbed water molecules and carboxylate groups originating from sodium acetate used in the synthesis.

In the rGO/CuFe_2_O_4_ composite spectrum, the C=C stretching peak is shifted to 1567 cm^−1^, indicating an interaction between CuFe_2_O_4_ and GO, likely due to bonding between Cu/Fe and the oxygen-containing functional groups on GO. The characteristic Fe–O peaks from CuFe_2_O_4_ are retained in the composite at 568 cm^−1^ and 424 cm^−1^, confirming the incorporation of CuFe_2_O_4_ into the GO matrix. Notably, the intensity of the peaks corresponding to C=O and O–H groups in GO is significantly reduced in the composite due to the strong reducing capability of ethylene glycol.

**Fig. 4:**
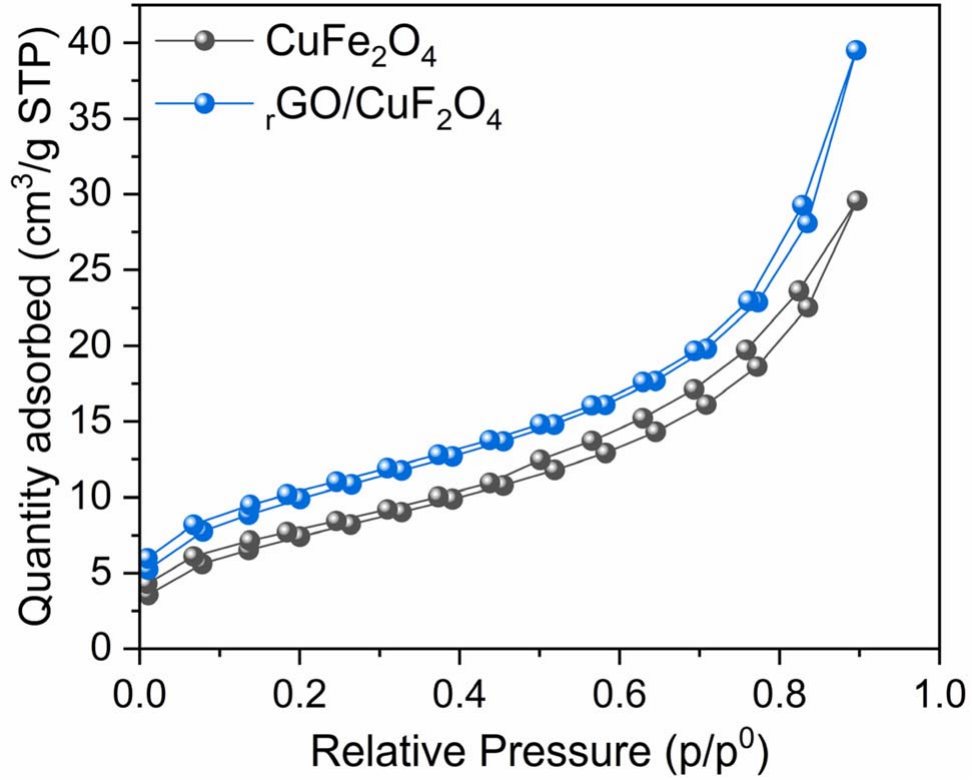
Nitrogen adsorption/desorption isotherm of CuFe_2_O_4_ and rGO/CuFe_2_O_4_ samples.

The nitrogen adsorption–desorption isotherms of CuFe_2_O_4_ and rGO/CuFe_2_O_4_ are presented in Fig. 4. Both samples exhibit a type IV isotherm with a distinct hysteresis loop according to the IUPAC classification, indicative of mesoporous structures. At a relative pressure of approximately 0.9, the quantity of nitrogen adsorbed by the rGO/CuFe_2_O_4_ composite reaches nearly 40 cm^3^ g^−1^, significantly higher than the 29 cm^3^ g^−1^ for CuFe_2_O_4_. The observed increase in surface area could be a key factor contributing to the enhanced catalytic activity of the _r_GO/CuFe_2_O_4_ composite, as discussed in detail later.

**Fig. 5:**
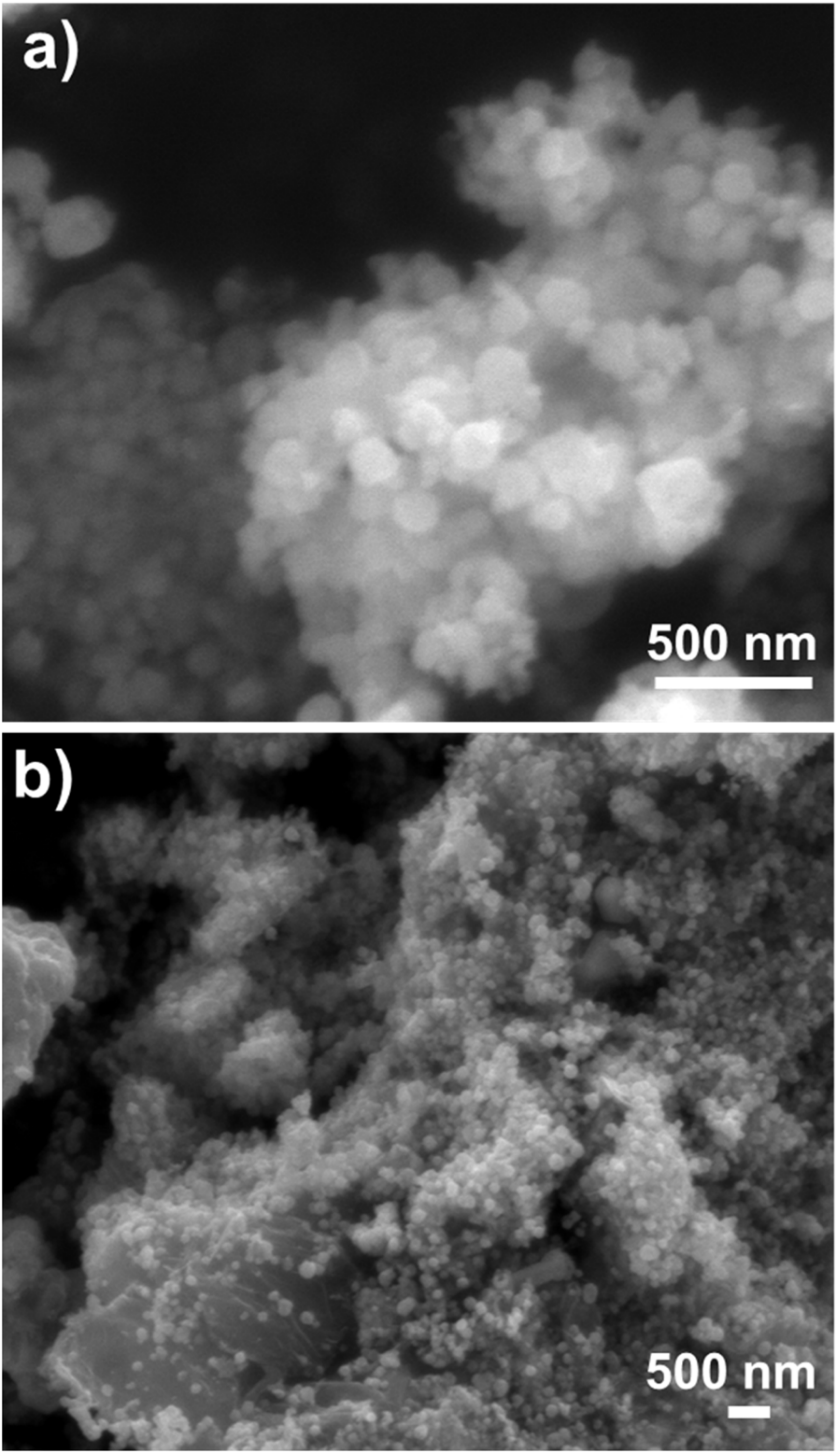
SEM images of (a) CuFe_2_O_4_ and (b) rGO/CuFe_2_O_4_ samples.

The surface morphology of CuFe_2_O_4_ and rGO/CuFe_2_O_4_ composites was examined using scanning electron microscopy (SEM) and transmission electron microscopy (TEM). The SEM images of CuFe_2_O_4_ particles (Fig. 5a) show agglomerated nanoparticles that are spherical and uniform in both shape and size. In contrast, the rGO/CuFe_2_O_4_ composite (Fig. 5b) exhibits a more dispersed morphology, with CuFe_2_O_4_ nanoparticles uniformly distributed across the rGO sheets. This uniform distribution prevents agglomeration, indicating a strong interaction between the CuFe_2_O_4_ nanoparticles and the rGO matrix.

**Fig. 6:**
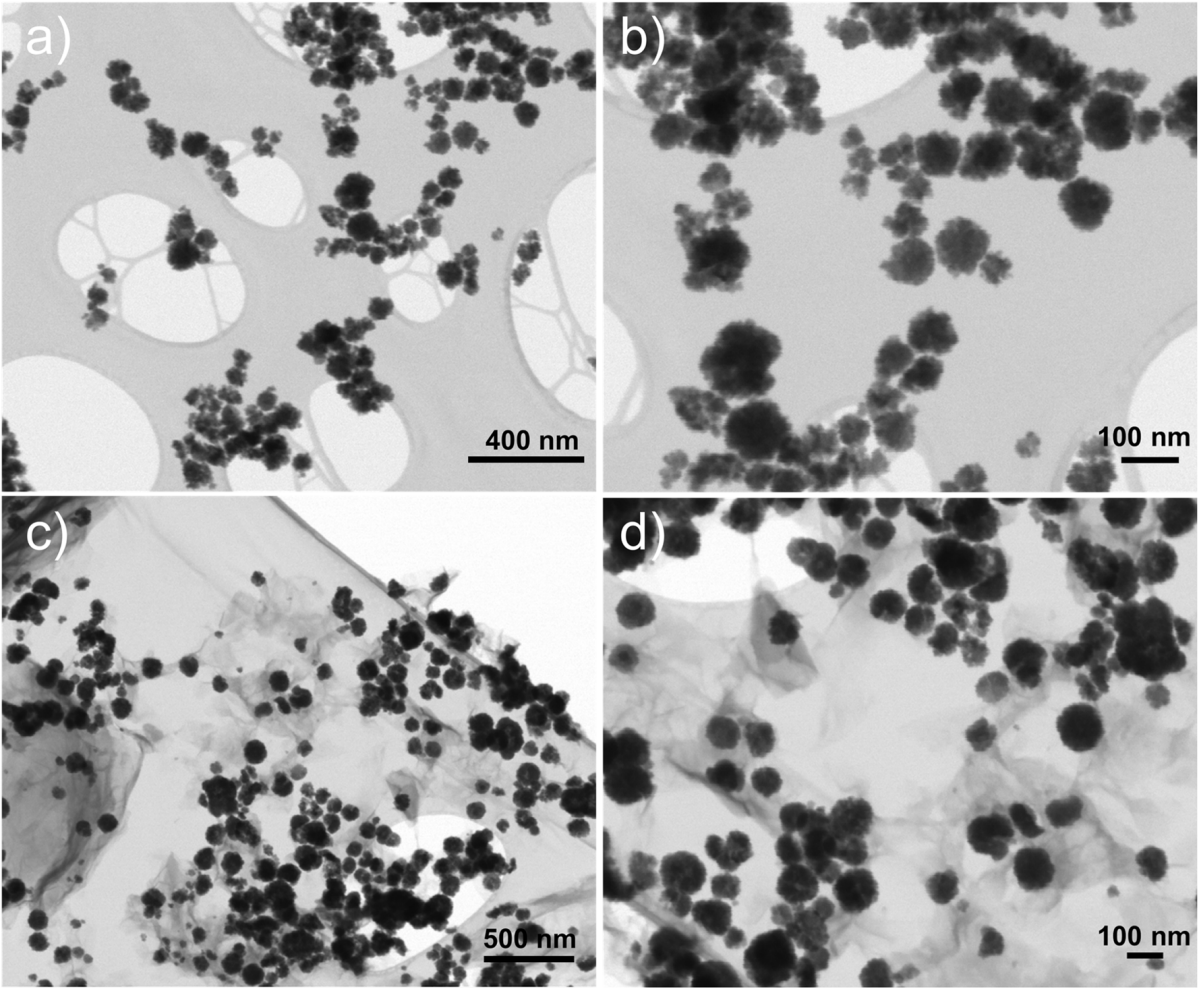
STEM images of (a and b) CuFe_2_O_4_ and (c and d) rGO/CuFe_2_O_4_ samples.

**Fig. 7:**
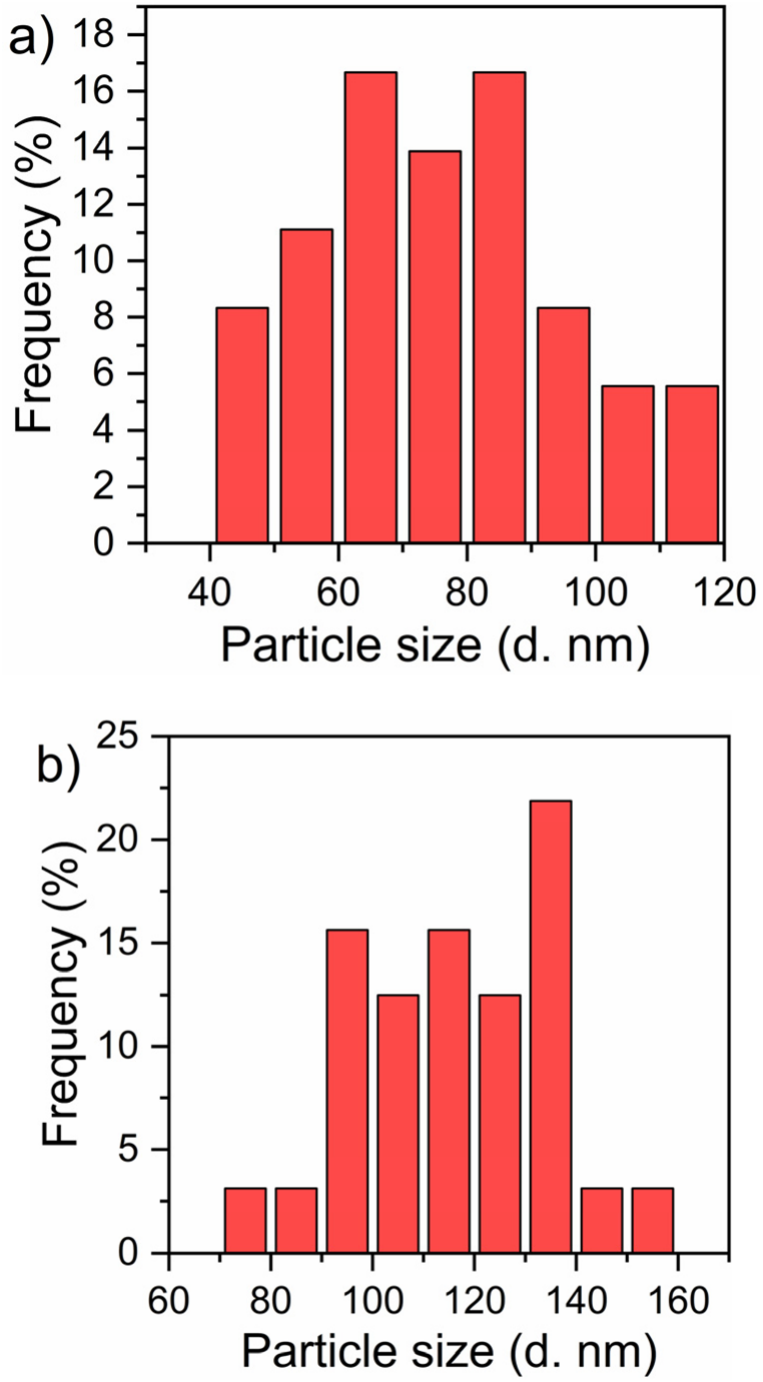
Particle size distribution analysis of (a) CuFe_2_O_4_ and (b) rGO/CuFe_2_O_4_.

The STEM image of CuFe_2_O_4_ (Fig. 6a and b) reveals the formation of well-dispersed, quasi-spherical nanoparticles with an average diameter of 82 nm, as shown in Fig. 7a. The particles exhibit a tendency to aggregate, which can be attributed to the magnetic properties of CuFe_2_O_4_. The STEM image of the rGO/CuFe_2_O_4_ nanocomposite (Fig. 6c and d) shows CuFe_2_O_4_ nanoparticles embedded within a reduced graphene oxide (rGO) matrix. The CuFe_2_O_4_ nanoparticles appear as dark spots, while the lighter regions represent the thin, sheet-like rGO layers. The rGO sheets act as a dispersing medium, preventing significant aggregation of the CuFe_2_O_4_ nanoparticles, as observed in the image. The nanoparticles remain well-distributed across the rGO surface, with fewer instances of particle clustering compared to the pure CuFe_2_O_4_ sample. In addition, the rGO/CuFe_2_O_4_ composite reveals a distinct difference in particle size compared to the pure CuFe_2_O_4_ sample. In this composite, the CuFe_2_O_4_ nanoparticles exhibit an increase in size with a mean diameter of 120 nm, as shown in Fig. 7b. This increase in particle size can be attributed to the interaction with the rGO sheets during the synthesis process, which may have facilitated further growth of nanoparticles on the rGO surface.

### 2.6 Catalytic activity of prepared materials

Orange G (OG), a synthetic dye widely present in industrial wastewater, was selected as a model pollutant to evaluate the catalytic efficiency of our materials in activating peroxymonosulfate (PMS). The degradation kinetics were monitored by measuring the decrease in OG concentration over time using UV-vis spectroscopy, specifically by tracking the attenuation of its characteristic absorption peak at 484 nm. The degradation efficiency was evaluated across different systems under identical experimental conditions (initial pH unadjusted).

**Fig. 8:**
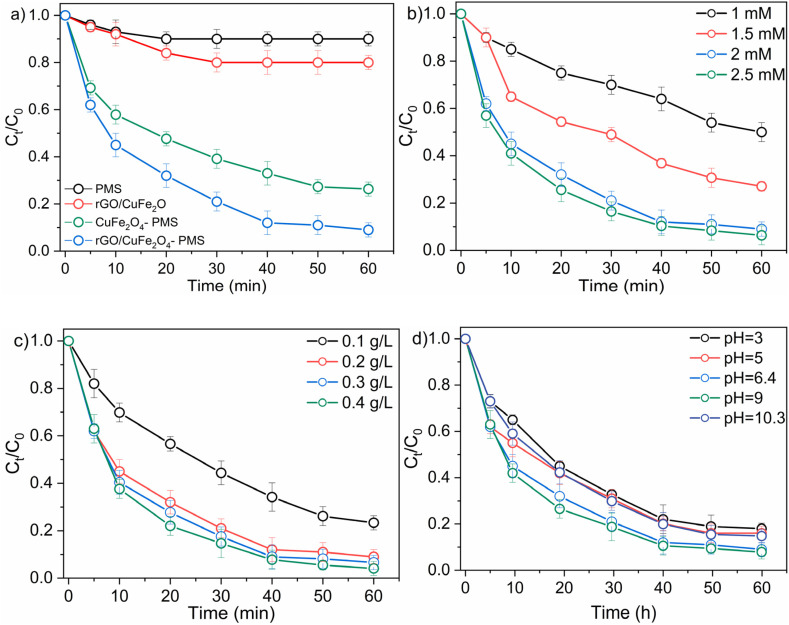
(a) Removal efficiency of OG in different system, Reaction conditions: [catalyst] = 0.2 g L^−1^; [PMS] = 2 mM; [OG] = 50 mg L^−1^; initial pH without adjustment. Effects of operating parameters on OG degradation (b) PMS dosage, (c) catalyst dosage, and (d) various pH conditions.

As illustrated in Fig. 8a, individual components—PMS alone (10% removal) and rGO/CuFe_2_O_4_ (19.6% removal)—exhibited limited degradation efficiency, indicating their poor catalytic activity toward OG when used independently. In contrast, CuFe_2_O_4_–PMS system showed significantly enhanced efficiency (73.6% removal). Remarkably, the ternary system (rGO/CuFe_2_O_4_–PMS) achieved the highest degradation efficiency (90.8%), within 60 minutes, attributed to the synergistic effects of improved adsorption on graphene oxide sites and enhanced radical generation from PMS activation. The exceptional catalytic performance of the rGO/CuFe_2_O_4_–PMS system can be explained by its effective activation of PMS to generate highly reactive radicals (SO_4_˙^−^ and ˙OH) and non-radicals (^1^O_2_) in the system. These powerful oxidizing species drive an efficient advanced oxidation process that rapidly decomposes OG molecules through radical-mediated reactions.

To evaluate the catalytic efficiency of the rGO/CuFe_2_O_4_–PMS system in OG degradation, we systematically investigated key operational parameters including PMS dosage, catalyst loading, and solution pH.

As shown in Fig. 8b, increasing PMS concentration from 1 mM to 2.5 mM substantially improved OG degradation efficiency (50.2% to 93.6%), and reaction kinetics (rate constant increased from 0.0112 to 0.0519 min^−1^). Notably, the enhancement was most pronounced between 1 and 2 mM, where *k* increased sharply from 0.0112 to 0.046 min^−1^. Beyond 2 mM, further PMS increase to 2.5 mM yielded only a modest kinetic improvement (0.046 to 0.0519 min^−1^), indicating approaching system saturation. This concentration-dependent behavior confirms that while higher PMS doses promote greater ROS generation and degradation efficiency, excessive concentrations (>2 mM) provide diminishing returns. This can be attributed to the fact that excessive addition of PMS generates a large number of free radicals, which compete with the target organic compounds.^41^ This competition triggers quenching reactions, thereby preventing any further enhancement in degradation efficiency. Considering both kinetic performance and economic factors, 2 mM was identified as the optimal PMS dosage for subsequent experiments.

The influence of different catalyst dosages on OG degradation using the rGO/CuFe_2_O_4_–PMS system was investigated. According to Fig. 8c, increasing the catalyst dosage from 0.1 to 0.2 g L^−1^ significantly enhanced the OG degradation efficiency, rising from 76.65% to 90.2% after 60 min. Correspondingly, the reaction rate constant increased from 0.026 to 0.047 min^−1^. This improvement is attributed to the greater number of active sites available at higher catalyst dosages, which promoted more efficient generation of reactive oxygen species and accelerated OG degradation. However, when the dosage reached 0.2 g L^−1^, the increase in OG removal rate began to level off, indicating that further increases provided only marginal benefits. Therefore, 0.2 g L^−1^ was selected as the optimal catalyst dosage for subsequent experiments, balancing degradation performance and material efficiency.

The performance of the rGO/CuFe_2_O_4_–PMS system in degrading OG was evaluated across a broad pH range (3–10.3) to investigate the influence of pH on catalytic efficiency. The natural pH of the OG solution was 6.4, unless otherwise specified, all experiments were conducted at this intrinsic pH. As shown in Fig. 8d, the catalyst demonstrated effective OG degradation across the entire pH range, with the highest performance observed at pH 9.2, followed by pH 6.4.

The reduced degradation observed under strongly acidic conditions (*e.g.*, 74.4% at pH 3 and 84% at pH 5 after 60 min) can be attributed to two key factors: (i) at low pH, excess hydrogen ions (H^+^) form strong hydrogen bonds with the peroxo (O–O) bond in PMS, stabilizing the molecule and thereby hindering its activation into reactive radicals; and (ii) PMS speciation is pH-dependent. While the reactive anionic form (HSO_5_^−^) predominates at pH value above ∼4, the molecular form H_2_SO_5_ dominates under highly acidic conditions, reducing the formation of sulfate and hydroxyl radicals and consequently lowering the degradation efficiency.^42^

**Fig. 9:**
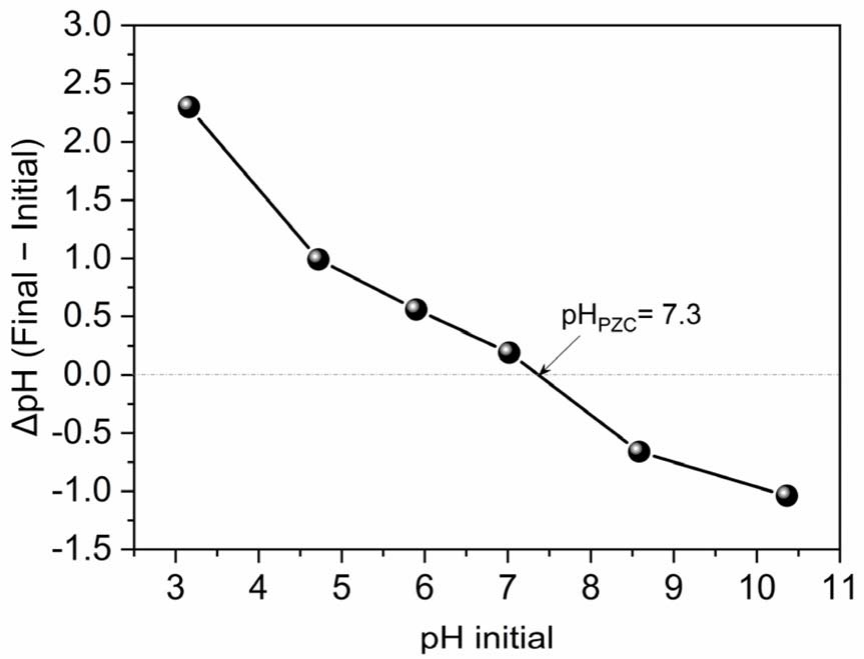
pH point zero charge (pH_PZC_) of rGO/CuFe_2_O_4_.

The point of zero charge (pH_PZC_) of the rGO/CuFe_2_O_4_ catalyst was determined to be 7.3 (Fig. 9). Below this pH, the catalyst surface is positively charged, which favors the electrostatic attraction of the anionic OG dye (deprotonated due to its high p*K*_a_ of 12.8). Conversely, at pH > 7.3, the catalyst surface becomes negatively charged, potentially causing electrostatic repulsion with the anionic dye. Nonetheless, the system exhibited significantly enhanced catalytic activity at pH 9.2. This improved performance may be due to two factors: first, the higher pH facilitates the deprotonation of PMS, increasing the concentration of the reactive HSO_5_^−^ species; second, alkaline conditions may promote secondary reactions leading to the generation of additional ROS, such as sulfate and hydroxyl radicals, thereby boosting overall degradation efficiency despite potential electrostatic repulsion. At a strongly alkaline pH of 10.3, no significant decrease in degradation efficiency was observed. However, it is important to note that the second p*K*_a_ of PMS is approximately 9.4, beyond which PMS undergoes further deprotonation to form SO_5_^2−^.^43^ This species exhibits lower oxidative potential, which may explain the decline in both degradation performance and reaction kinetics under such conditions.

**Fig. 10:**
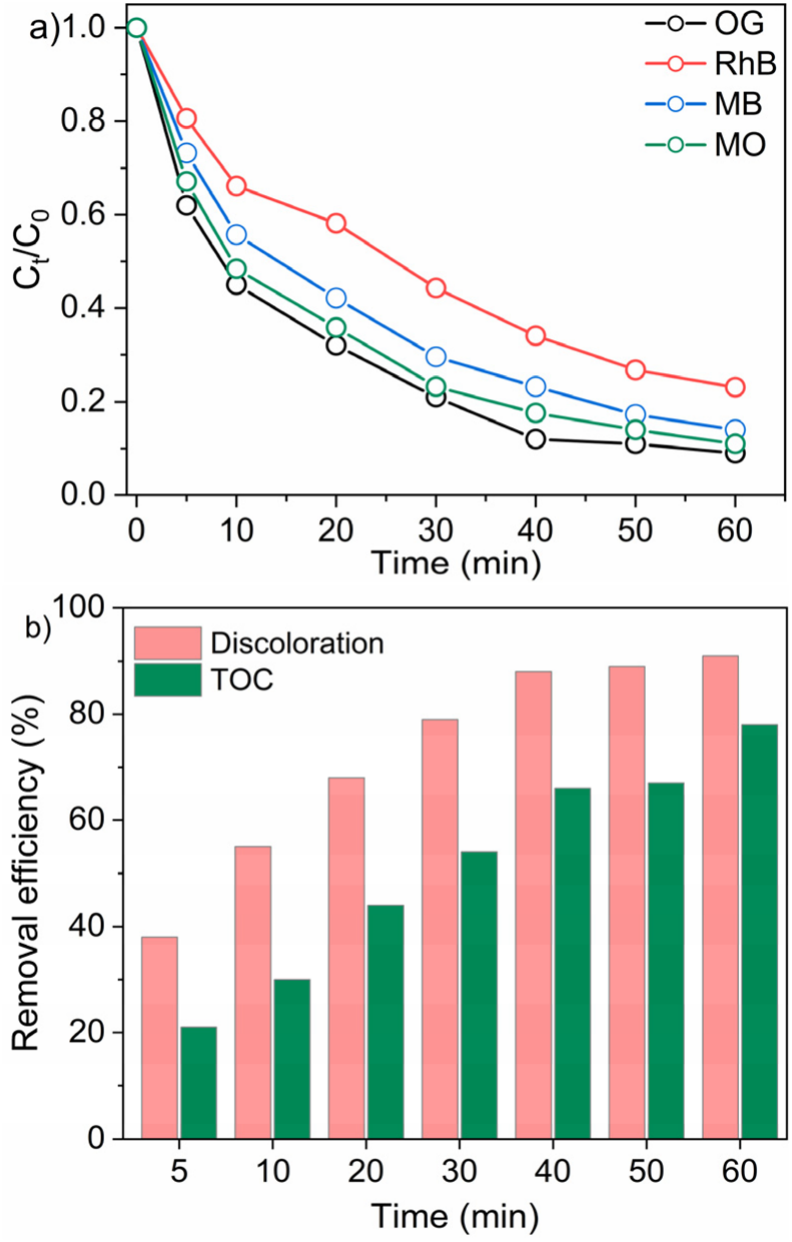
(a) Degradation of various organic dyes using the rGO/CuFe_2_O_4_–PMS system. (b) Discoloration and mineralization (TOC removal) of OG at different reaction times under optimal conditions. Reaction conditions: [catalyst] = 0.2 g L^−1^; [PMS] = 2 mM; [OG] = 50 mg L^−1^, [BM] = 10 mg L^−1^; [RhB] = 10 mg L^−1^; [MO] = 10 mg L^−1^.

Fig. 10a illustrates the comparative degradation performance of various organic dyes (OG, RhB, MB, and MO) using the rGO/CuFe_2_O_4_–PMS catalytic system over a period of 60 minutes. Among the tested pollutants, OG exhibited the most rapid degradation, maintaining the lowest relative concentration (*C*_*t*_/*C*_0_) throughout the reaction time. After 60 minutes of treatment, the decolorization efficiencies for RhB, MB, and MO reached 76.8%, 86.1%, and 89.0%, respectively. These results demonstrate that the rGO/CuFe_2_O_4_–PMS system effectively activates PMS to degrade a broad range of organic contaminants, highlighting its excellent catalytic versatility and potential for practical wastewater treatment applications.

Fig. 10b provides further insight into the degradation performance of OG by comparing discoloration and total organic carbon (TOC) removal. Discoloration exceeded 91% within 60 minutes, reflecting the effective cleavage of the chromophoric structures responsible for OG's visible color. However, the TOC removal—an indicator of complete mineralization of organic pollutants into CO_2_ and H_2_O—reached approximately 78% over the same duration. This highlights the ability of the rGO/CuFe_2_O_4_–PMS system to achieve not only rapid decolorization but also significant mineralization within the treatment period. A comparative analysis of different catalysts for organic dye degradation *via* PMS activation is presented in Table 1. In summary, the proposed rGO/CuFe_2_O_4_ system demonstrates competitive performance for the removal of organic pollutants, comparable to other reported materials, confirming its potential as an effective Fenton-like catalyst.

**Table 1:** Comparison of organic pollutants degradation *via* Fenton-like PMS activation

Catalyst	Pollutant conc. (mg L^−1^)	Catalyst dose (g L^−1^)	PMS conc. (mM)	Time (min)	Removal efficiency (%)	Ref.
NH_2_OH/Fe_3_O_4_	[OG] = 45	0.5	1.0	30	99	44
NH_2_-MIL-101(Fe)	[OG] = 50	0.2	1.0	60	98	45
MnFe_2_O_4_/α-MnO_2_	[OG] = 50	0.1	3.25	30	97	46
CuMg oxide/g-C_3_N_4_	[RhB] = 10	0.3	1.0	5	99	47
α-MnO_2_ nanowires	[RhB] = 20	0.2	0.16	30	95	48
rGO/CuFe_2_O_4_	[OG] = 50	0.3	2.0	60	91	This work
	[RhB] = 10				78	

**Fig. 11:**
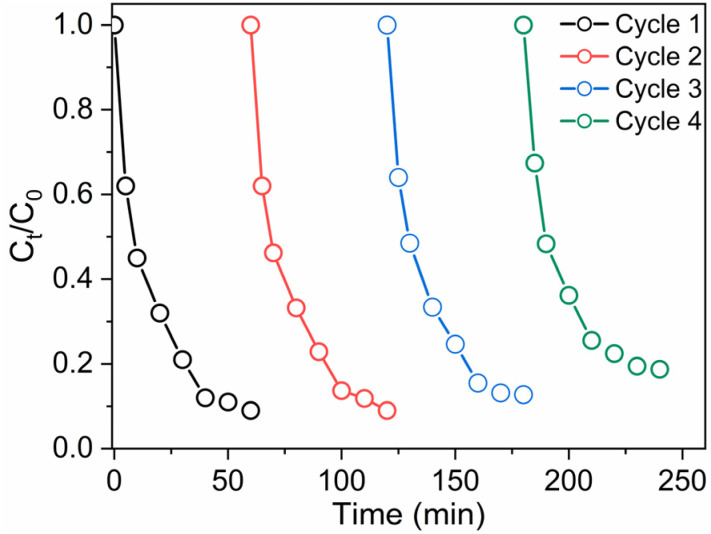
Recycling tests of the rGO/CuFe_2_O_4_ catalyst; reaction conditions: [catalyst] = 0.2 g L^−1^; [PMS] = 2 mM; [OG] = 50.

When applying catalytic AOPs for pollutant degradation, the reusability and long-term stability of the catalyst are critical for practical implementation. To evaluate the recyclability of the rGO/CuFe_2_O_4_ nanocatalyst, four consecutive degradation cycles were performed using the same catalyst to activate PMS for the degradation of OG. As depicted in Fig. 11, the rGO/CuFe_2_O_4_ catalyst exhibited consistent catalytic performance across all four cycles. The degradation curves show that the catalyst maintained nearly identical activity throughout the cycles, with minimal performance loss. These results confirm the excellent stability and reusability of the rGO/CuFe_2_O_4_ system, making it a promising candidate for practical wastewater treatment applications. Leaching of Cu^2+^ ions was evaluated in the solution after the first cycle using ICP-MS analysis. A low concentration of 0.13 mg L^−1^ was detected, which complies with European discharge standards, indicating that the catalytic sites in the rGO–CuFe_2_O_4_ catalyst remain stable during PMS activation.

### 2.7 Identification and contribution of reactive oxygen species (ROS)

**Fig. 12:**
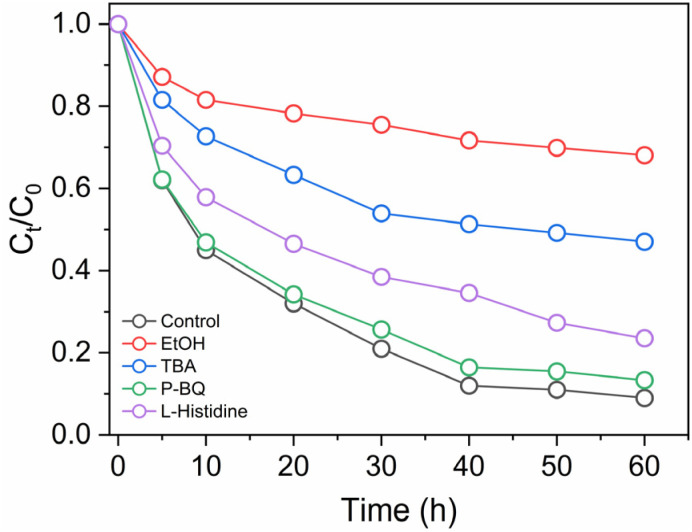
The effect of scavengers on OG degradation using different scavengers.

To elucidate the degradation mechanism of orange G in the rGO–CuFe_2_O_4_/PMS system, quenching experiments were performed using various scavengers, and the results are illustrated in Fig. 12. Each quencher targets specific ROS, enabling the estimation of their respective roles in the oxidation process. Ethanol (EtOH) is frequently used to quench both SO_4_˙^−^ (rate constant *k* ≈ 10^7^ M^−1^ s^−1^) and ˙OH (*k* ≈ 10^9^ M^−1^ s^−1^), while *tert*-butyl alcohol (TBA) selectively scavenges ˙OH (*k* ≈ 6.0 × 10^8^ M^−1^ s^−1^). Additionally, *p*-benzoquinone (*p*-BQ) and l-histidine are widely used as scavengers for superoxide radicals (O_2_˙^−^, *k* ≈ 9 × 10^8^ M^−1^ s^−1^) and singlet oxygen (^1^O_2_, *k* ≈ 10^9^ M^−1^ s^−1^), respectively.^49–52^

In the absence of scavengers (control), the system achieved a degradation efficiency of 90% after 60 minutes, confirming the high catalytic activity. The addition of EtOH, which scavenges both ˙OH and SO_4_˙^−^, significantly suppressed degradation to 32%, indicating a combined radical contribution of approximately 58%.

When TBA was used to selectively quench ˙OH, the degradation dropped to 53.1%, suggesting a ˙OH contribution of 36.9%. By subtracting this from the total inhibition by EtOH, the SO_4_˙^−^ radical contribution is estimated at 21.1%. The presence of *p*-BQ, a scavenger for O_2_˙^−^, resulted in 86.7% degradation, indicating a relatively low O_2_˙^−^ contribution of 3.3%. l-Histidine, which quenches ^1^O_2_, achieved a degradation efficiency of 76.4%, corresponding to a ^1^O_2_ contribution of 13.6%.

This result confirms the coexistence of both radical and nonradical oxidation pathways in the rGO–CuFe_2_O_4_/PMS system, contributing synergistically to the overall degradation efficiency.

**Fig. 13:**
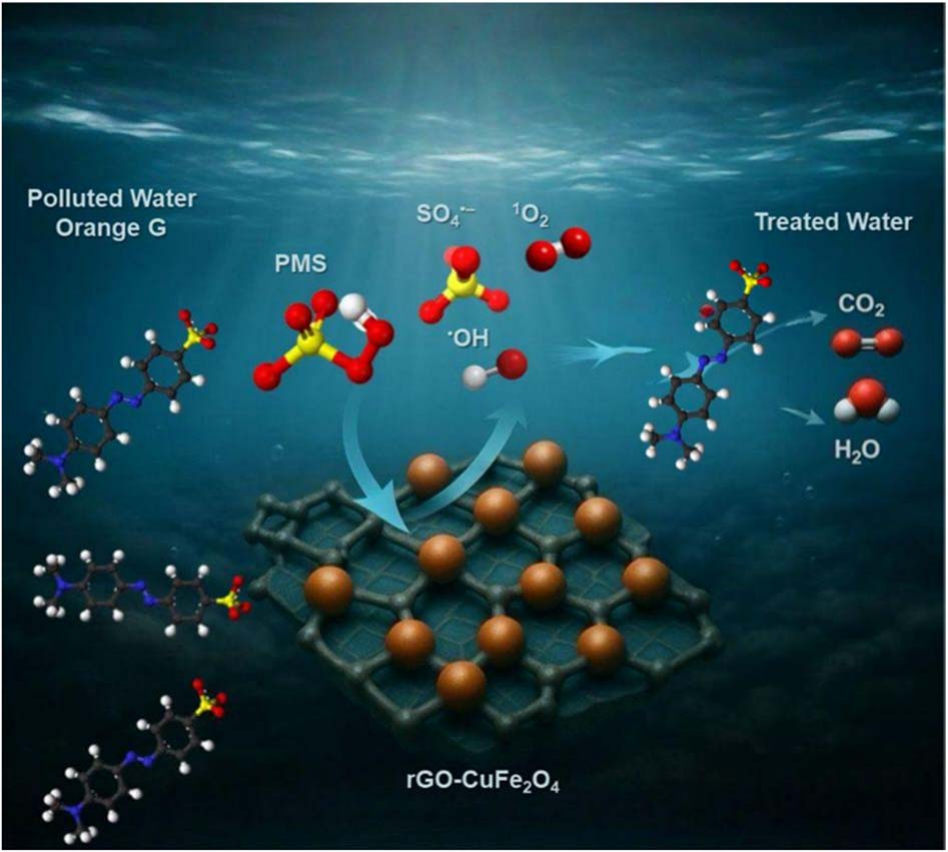
Proposed mechanism of orange G degradation in the rGO–CuFe_2_O_4_/PMS system.

As shown in Fig. 13, the degradation of OG in the rGO/CuFe_2_O_4_/PMS system is driven by both radical and non-radical oxidative pathways, as supported by the quenching experiments. Upon addition of PMS, Cu^2+^ species present on the catalyst surface act as primary activators. The Cu^2+^ ions can undergo two redox processes with PMS, either being oxidized to Cu^3+^ or reduced to Cu^+^, resulting in the generation of SO_4_˙^−^ radicals:

1 Cu^2+^ + HSO_5_^−^ → Cu^3+^ + SO_4_˙^−^ + OH^−^

2 Cu^2+^ + HSO_5_^−^ → Cu^+^ + SO_4_˙^−^ + H^+^

The resulting Cu^+^ species can be reoxidized by PMS, maintaining the redox cycle and continuously generating SO_4_˙^−^:

3 Cu^+^ + HSO_5_^−^ → Cu^2+^ + SO_4_˙^−^ + OH^−^

Simultaneously, Cu^3+^ may react with PMS to form SO_5_˙^−^ radicals:

4 Cu^3+^ + HSO_5_^−^ → Cu^2+^ + SO_5_˙^−^ + H^+^

However, given the lower oxidative potential of SO_5_˙^−^ (*E*° ≈ 1.1 V), its contribution to OG degradation is considered negligible. The dominant reactive species, SO_4_˙^−^, directly attacks OG molecules:

5 SO_4_˙^−^ + OG → intermediate + CO_2_ + H_2_O

Additionally, SO_4_˙^−^ reacts with water, generating hydroxyl radicals (˙OH), thereby enhancing the overall oxidative capacity:

6 SO_4_˙^−^ + H_2_O → SO_4_^2−^ + H^+^ + ˙OH

These newly formed ˙OH radicals also contribute to OG degradation *via*:

7 ˙OH + OG → intermediate + CO_2_ + H_2_O

In addition to these radical pathways, the presence of ^1^O_2_ as a non-radical oxidant is also inferred. This species is likely generated through PMS activation at the surface of rGO–CuFe_2_O_4_*via* electron transfer mechanisms. The conductive rGO support enhances charge transport between the Cu^2+^/Cu^+^ and Cu^2+^/Cu^3+^ redox couples, sustaining continuous PMS activation and facilitating multiple oxidative pathways.

## 3. Conclusion

A magnetic catalyst based on reduced graphene oxide-supported CuFe_2_O_4_ was successfully synthesized *via* a solvothermal method and demonstrated excellent performance in PMS activation for orange G degradation. Characterization confirmed the presence of well-dispersed CuFe_2_O_4_ nanoparticles on rGO sheets, resulting in increased surface area and minimized particle aggregation. Under optimal conditions, the rGO/CuFe_2_O_4_ catalyst achieved over 90% removal of orange G and 78% TOC reduction. Moreover, the catalyst exhibited broad pH applicability, high stability, and reusability over multiple cycles. Importantly, it also showed versatile catalytic activity by effectively degrading other dyes such as rhodamine B (78%), methylene blue (86%), and methyl orange (89%). These findings highlight the potential of rGO/CuFe_2_O_4_ as a cost-effective, efficient, and environmentally friendly material for advanced oxidation processes in wastewater treatment targeting a wide range of organic pollutants.

## Conflicts of interest

There are no conflicts to declare.

## Data Availability

All data supporting the findings of this study are available within the main manuscript.
