# Correction: Copper ferrite–graphene oxide catalyst for enhanced peroxymonosulfate activation and pollutant degradation

**DOI:** 10.1039/d6na90040b

**Published:** 2026-06-08

**Authors:** Imane Sebah

**Affiliations:** a Laboratory of Organic Chemistry and Physical Chemistry (Molecular Modeling and Environment), Faculty of Sciences, University Ibn Zohr Agadir Morocco Imane.sabah@outlook.fr

## Abstract

Correction for ‘Copper ferrite–graphene oxide catalyst for enhanced peroxymonosulfate activation and pollutant degradation’ by Imane Sebah, *Nanoscale Adv.*, 2025, **7**, 5646–5657, https://doi.org/10.1039/d5na00409H.

The author regrets they included Moustapha Belmouden as an author without their knowledge or permission. Moustapha Belmouden has been removed from this paper. In addition, the email address for the author Imane Sebah was incorrect in the original article; it is corrected here.

The Royal Society of Chemistry apologises for these errors and any consequent inconvenience to authors and readers.

